# Measuring bereavement prevalence in a complex sampling survey: the 2019 Georgia Behavioral Risk Factor Surveillance System (BRFSS)

**DOI:** 10.1186/s12874-023-01917-5

**Published:** 2023-06-13

**Authors:** Changle Li, Toni P. Miles, Ye Shen, Rana Bayakly, Moses Ido, M. Mahmud Khan

**Affiliations:** 1 Department of Epidemiology and Biostatistics, University of Georgia, Athens, USA; 2 Georgia Department of Public Health, Atlanta, USA

**Keywords:** BRFSS, Bereavement, Grief, Aging, Population Surveillance, Weighting, Multiple Imputation, Public Health

## Abstract

**Background:**

The Behavioral Risk Factor Surveillance System (BRFSS) is an annual survey designed to identify trends in the public’s health. In its 2019 field survey, the U.S. state of GA tested a new 3 – item module to measure the numbers of bereaved, resident adults aged 18 years and older. Participants were eligible if they answered ‘Yes’ to the item ‘Have you experienced the death of a family member or close friend in the years 2018 or 2019?’. This analysis explores two research questions. Can estimates for bereavement prevalence be derived without large sampling errors, low precision, and small subsamples? Can multiple imputation techniques be applied to overcome non-response and missing data to support multivariate modeling?

**Methods:**

BRFSS is a non-institutionalized sample of adults aged 18 years and older living in the U.S. state of Georgia. Analyses in this study were conducted under two scenarios. Scenario 1 applies the complex sample weights created by the Centers for Disease Control and imputes values for missing responses. Scenario 2 treats the data as a panel – no weighting combined with removal of persons with missing data. Scenario 1 reflects the use of BRFSS data for public health and policy, while Scenario 2 reflects data as it is commonly used in social science research studies.

**Results:**

The bereavement screening item has a response rate (RR) of 69.1% (5206 of 7534 persons). Demographic subgroups and categories of health have RR of 55% or more. Under Scenario 1, the estimated prevalence of bereavement is 45.38%, meaning that 3,739,120 adults reported bereaved in 2018 or 2019. The estimated prevalence is 46.02% with Scenario 2 which removes persons with any missing data (4,289 persons). Scenario 2 overestimates the bereavement prevalence by 1.39%. An illustrative logistic model is presented to show the performance of exposure to bereavement under the two data scenarios.

**Conclusions:**

Recent bereavement can be ascertained in a surveillance survey accounting for biases in response. Estimating bereavement prevalence is needed for measuring population health. This survey is limited to one US state in a single year and excludes persons aged 17 years and younger.

**Supplementary Information:**

The online version contains supplementary material available at 10.1186/s12874-023-01917-5.

## Introduction

Bereavement is a known risk factor for morbidityand mortality. By taking a social network view, researchers have documented a broader circle of persons who are connected to a single death [1–5]. One recent study has created a bereavement multiplier. This multiplier is based on analyses of single deaths within a kinship network and estimates that nine persons on average are connected to a single Covid-19 death [6]. The Kinship risk multiplier analysis is helpful because it provides an estimate of the numbers of persons bereaved. Kinship risk operates like flood risk analysis. Flood risk identifies the number of buildings damaged by a single flood event [7] while kinship risk identifies the numbers of persons in the social network of a singular decedent. Is kinship risk, i.e., bereavement prevalent enough to merit its inclusion in an ongoing surveillance survey? To answer this question, the U.S. state of Georgia field tested a new bereavement module in its 2019 BRFSS field survey. The product of this effort is an estimate of the number of persons responding ‘Yes’ to a query about bereavement in a defined time frame. The analyses presented here tests the feasibility of surveillance for bereavement. Are there enough persons bereaved within a similar time frame to support the assessment of bereavement-related injury?

Currently, societal risk due to bereavement is indirectly inferred from big data sources such as population registries [8], or complex sampling surveys [9, 10]. The Swedish population registry is one source for measuring survivor mortality after the death of a family member [8]. The Swedish registry has the capacity to link family members alive or dead. The National Mortality Followback Survey (NMFS) is a complex sampling survey of death certificates in the U.S [9]. NMFS is designed to validate death certificates and ascertain events surrounding decedent health in the 3 months prior to the death. Data are obtained by interviewing key informants, usually a family member. Despite the sensitive nature of the topic, participation rates in NMFS ranged from 90 to 95% in the 3 cycles – 1966, 1986, and 1993. NMFS does not have data on the informant other than age, gender, and race. The Health and Retirement Survey (HRS) is a longitudinal complex sampling survey of U.S. adults aged 50 years and older in which cohort members are recontacted once at two-year intervals [10]. The HRS item on bereavement was introduced in 2006. Analyses of HRS data has been used to identify individual mediators and moderators of health related to bereavement and factors supporting resilience to negative health effects [2, 3, 11–13]. HRS respondents have a participation rate of 80%. Taken together, the evidence from both surveys indicates that participants are willing to respond when asked specific questions about the deaths of friends and family.

## Methods

### Sample

The 2019 Georgia BRFSS is a sample of non-institutionalized adults and consists of an unweighted panel of 7,354 responding to the common core items. A subset (*n* = 5,206) also responded to the recent bereavement module placed at the end of the interview. The core interview took an average of 17 min and the bereavement module added 5 min. Interviewees were recruited from list-assisted, random digit dialing of adults randomly selected from the non-institutionalized population aged 18 years and older resident in Georgia households drawn from within primary statistical units. Persons were recruited from both landline and cellular phones. For these analyses, the final panel included 4,289 respondents with complete information on all 15 core and module items. The missing responses were not uniform across individual items. See Additional file 1: Appendix Table A for the list of variables used in the analysis and rates of missing data for each. Results presented in the Tables and Figures are derived from either the panel or multiple imputation, weighted sample (MI). Methods for the creation of the MI sample are described in statistical methods.

### Measures

The common core contains uniform survey items asked in all U.S. states on health risk behaviors, chronic diseases, access to health care, and use of preventive services. The analytic dataset used in this study contains items from the following categories – Demographics (Age, Race / Ethnicity, Sexual Orientation / Gender Identity); Social Determinants (Education, Residence in Metropolitan Counties, Employment); Health Behaviors (Physical Activity, Smoking, Alcohol Use); and Quality of Life (Self rated health, Physical and Mental Health). The format for these items is described in detail elsewhere [14].

#### Recent bereavement

The 2019 Georgia BRFSS added a new state module on the topic of bereavement to the end of the interview. All participants were asked the question. The module contains questions from the HRS and have been described elsewhere [10].. *‘Have you experienced the death of a family member or close friend in the years 2018 or 2019*? were further queried about the number of losses and their relationship to each decedent. The data from the number of deaths and the relationships are the subject of a separate report.

#### Demographic variables: gender identity / sexual orientation, age, race / ethnicity

Binary gender, age, race, and ethnicity are a part of the common core questions asked by all states. The Georgia BRFSS also has three items asking about sexual orientation and gender identity. To define sexual orientation, participants were asked: *Which of the following best represents how you think of yourself? Do you consider yourself to be transgender*? Response options for self-identity include Gay, Straight or Bisexual or Something else. Response options for the transgender item include Transgender male to female, Transgender female to male, and transgender nonconforming. The survey also queries age (in years) and self-selected race and ethnicity from a series of U.S. census bureau categories. See Additional file 1: Appendix Table B for the Georgia BRFSS formulation of Sex and Gender Identity questions.

### Statistical methods

#### Response rates

Of the original 7,354 persons answering the BRFSS core questions, there were 5,206 persons from the original sample who responded to the bereavement module screening question for a response rate of 70.8%. Response rates for subgroups in the bereavement module are calculated by dividing the number of subgroup members completing an item by the total subgroup numbers in the module panel.

#### Multiple imputation

Prevalence of bereavement is a singular measure. In statistical models, missing responses to the other items used in the analyses become an increasingly important consideration. Multiple imputation is a simulation-based statistical technique that allows researchers to use more available data, thus reducing biases when persons with missing data are excluded [15, 16]. Multiple imputation has three elemental phases: imputation, analysis, and pooling. The imputation phase is to create m copies of the dataset, with the missing values replaced by imputed values using an appropriate model. Rubin suggested that m = 5 should be sufficient to obtain valid inference, while some researchers reported m should be 50 or more [17–20]. Missing data elements can stem from three possible situations: missing completely at random (MCAR), missing at random (MAR), and missing not at random (MNAR). MCAR occurs when the missingness is unrelated to the observed and unobserved value for that unit [21].Under a MAR mechanism, the probability of a missing value for an item may depend on observed data but not on unobserved data. MNAR means that the probability of missingness depends on the underlying value of an item [22].

Steps creating the final MI sample proceeded as follows. First, complex sampling weights were applied to the panel and the variables weighting, stratification, and primary sampling unit were used. The complex sampling weights were applied to the panel using the variable _llcpwt for weighting, _ststr for stratification, and the variable _psu for primary sampling unit [23]. Next, 50 copies of the weighted data were created. This number was chosen to reduce the sampling error due to imputations. The imputation process was then carried out based on multiple imputation by chained equations (MICE). The MICE method is a practical approach to impute missing data in multiple variables based on a set of univariate imputation models [20]. We selected the conditional models based on the type of variables. The MICE method allows the use of logistic regression model to impute binary variables such as bereavement. Moreover, ordered logistic and multinomial logistic regression models can impute ordered categorical such as educational attainment and unordered categorical variables such as race. The estimated variance of this MI estimate is calculated based on Rubin’s rules [21]. Standard imputation models with Rubin’s rules result in an upwardly biased estimate of the variance [24]. Rubin’s variance estimator combines the average of the variance estimates using complex sample variance estimates. Therefore, we included the sampling weights as a linear term in the imputation model [25]. The estimated variance of the MI-based estimate is calculated based on the between-imputation variance and the within-imputation variance.

Testing on whether the data set is MCAR was performed. Little’s MCAR test gives a χ^2^ distance of 1633.93 with the degree of freedom = 1052 and a *p*-value < 0.001 [26]. The test suggests that the missing data of the measured variables included the analyses are not MCAR. In this phase, each of the 50 complete datasets was used to calculate prevalence rates in the weighted sample. The results obtained from the 50 completed datasets are combined into a single multiple-imputation result in the pooling phase [20]. The single parameter estimate is the mean of the m (= 50) parameter estimates.

#### Prevalence estimates, relative difference, and standard errors

To see the biases created by missing data, prevalence rate estimates were calculated using both the panel sample and the MI sample. A single measure – the Relative Difference (RD)—is a ratio showing the relative difference of bereavement prevalence estimates as a percent difference between the two samples. The numerator is calculated by subtracting MI-based estimate from panel-based estimate and can have either a positive or negative value. The panel estimate is used as the denominator for this ratio. RD illustrates the effect on the estimated prevalence in a scenario where survey design and missing responses are ignored. The associated negative and positive signs provide direction for this difference. A negative RD means that panel data underestimates bereavement prevalence. A positive RD indicates that panel data overestimates prevalence. BRFSS is designed the development of population estimates by public health agencies. The preferred denominator for this application is the weighted MI estimate. The panel estimates maybe an acceptable substitute when exploring mechanisms.

These analyses also calculate standard errors associated with estimates derived from the panel and the weighted-MI data. Standard error indicates the uncertainty around each estimated rate. The standard error is also a component in calculating a confidence interval. The estimated SEs for the weighted-MI estimates account for biases associated with not using sample weights and not accounting for missing responses, hence SEs of weighted imputed estimates are larger.

The logistic regression demonstration included in this paper shows the confidence interval because it is designed for the development of public health reports. Although the results are not shown in these analyses, we explored two approaches for calculating the 95 percent confidence intervals for the estimates – the Clopper-Pearson (CP) method [27] and the Korn-Graubard (KG) adjustment [28] to the Clopper-Pearson method. In the analyses, we used the CP method for the panel data and the KG for complex survey data (i.e., the weighted survey data). These are used when the normal approximation to the binomial does not work well – typically when the expected number of cases (number in the numerator) is less than 5. This will occur for rare events and / or small samples. These methods produce non-symmetric confidence intervals. They would be relevant for estimates derived from sub-groups that have small numbers of events, i.e.5 or fewer people suffering bereavement – in this case roughly 5/0.46 = 12 or less.

The KG confidence interval was developed specifically for analyzing survey data with a complex design and uses weighted data without imputation. As anticipated, CP and KG yield similar confidence intervals. For the panel sample (4,289 respondents), the standard error uses the traditional approach available in statistical packages. The SE is an analytically derived variance estimator associated with the sample proportion. This approach ignores missing response and characteristics of the sample design. On the other hand, when BRFSS weights and imputed data are used to calculate an estimate, the SE is obtained based on Rubin’s rule. Rubin’s rule combines the average within imputation variance with the between imputation variance in estimates using complex sample variance estimates.

All statistical analyses were conducted using Stata Version 17 (StataCorp, College Station, TX). First, the *proportion* and *logistic* commands were used to calculate the SEs associated with the ORs from the logistic regression in the panel data. Second, the *svyset* command was used to account for complex sampling weights. Third, the key commands are *mi set*, *mi register imputed*, and *mi impute chained* commands for creating multiple imputation. Last, the *mi estimate: svy: proportion* and *mi estimate or: svy: logistic* commands were used to calculate the SEs for estimates associated with the ORs from the logistic regression model using the weighted-MI data set.

All models in Table 3 included the interaction terms which is represented as the product of two independent variables (Bereavement and Gender). The models also included the main effects for bereavement and gender. For example, when the dependent variable is current smoker, we can see the main effects (ORs) of bereavement and gender are 1.52 and 1.66, and the interaction effect is 0.74. To evaluate the risk of reporting a current smoker, the model is as follows:

$$\begin{array}{c}\mathbf{C}\mathbf{u}\mathbf{r}\mathbf{r}\mathbf{e}\mathbf{n}\mathbf{t}\mathbf{s}\mathbf{m}\mathbf{o}\mathbf{k}\mathbf{e}\mathbf{r}=[Main effects]\mathrm{ a}*\mathrm{bereavement }+\mathrm{ b}*\mathrm{gender }+[Interaction]\mathrm{ c}*\mathrm{bereavement}*\mathrm{gender }+ [controls]\mathrm{ d}*\mathrm{age }+\mathrm{ e}*\mathrm{race}\\ \mathrm{a},\mathrm{ b},\mathrm{ c},\mathrm{ d},\mathrm{ and e are coefficients}\end{array}$$

## Results

**Fig. 1:**
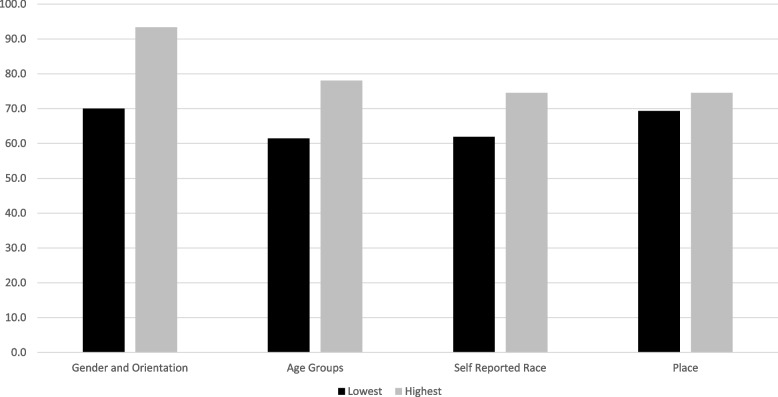
Categorical Response Rates to the Bereavement item, 2019 Georgia BRFSS

Are participants willing to discuss the deaths of family and friends? Fig. 1 shows response rates to the question *‘*Have you experienced the death of a family member or friend in the years 2018 or 2019?’. The response rates shown in Panel C and D reflect the proportion of module participants (*n* = 5206) who also answered the specific item. There were 4,289 participants who had complete information on all 15 items. The remaining 917 were missing one or more responses and were deleted from the panel data. Additional file 1: Appendix Table A shows the missing response rates for each individual item. For example, the response rate for gender is 100% and the response rate for the 1st bereavement item is 70.71%. Men (69.99%) are less likely to respond to the bereavement item while women (71.42%) are more likely to respond. The smoking item has a response rate of 93.11% (*N* = 6,847). Persons who answer the smoking item answer the bereavement item were very similar – 75.38% of ‘No’ and 75.86% of ‘Yes’. Response biases within categories was a first strategy to evaluate survey data on bereavement.

Table 1 compares the estimated rates of bereavement in the panel with the MI samples. The rate for the MI sample is 45.38% on a base population of 8,164,018 adults aged 18 years and older. By using this rate, an analyst can project that there are 3,739,120 adults who reported the death of at least one family member or friend in 2018 or 2019. Across the MI subgroups, estimates rates range from 37.4% to 55.7%. In the panel sample, the population prevalence increases to 46.02% with a range of 38.51% to 54.98%. The column labeled RD (Relative Difference) is a ratio showing the relative difference of the estimate as a percent difference between the two samples. The RD illustrates the effect of ignoring survey design and deleting persons with missing responses. The RD of 1.39% means that panel data overestimates the MI rate. Each subgroup’s estimated rate and their associated RD are shown in the table. The absolute value of RD increases with subgrouping. While the relative differences are often less than 5%, there are some that are larger. These larger RDs are observed for the subgroups – SOGI All Other (7.82%), Race All Other (10.36%), and Unemployed (7.65%). The larger RDs show that weighting and MI reduce biases affecting panel-based estimates for these vulnerable groups.

**Table 1:** Prevalence of Bereavement and Relative Difference, Panel versus MI data. 2019 Georgia Behavioral Risk Factor Surveillance System (BRFSS)

	**Panel** *N* = 4,289	**Weighted MI** *N* = 8,164,018	**Relative Difference (RD, %)**
**Percent reporting bereavement**	46.02	45.38	1.39
**Demographics**			
Males	43.89	44.24	-0.80
Females	47.66	46.43	2.58
SOGI^**§**^: CIS Gender	46.88	45.71	2.50
SOGI^**§**^: All other	44.48	41.00	7.82
18 – 24 years	38.51	37.49	2.65
25 – 34 years	42.60	43.60	-2.35
35 – 44 years	48.41	47.77	1.32
45 – 54 years	48.20	48.01	0.39
55 – 64 years	48.66	50.15	-3.06
65 + years	45.14	43.85	2.86
Black / African American only, NH	54.98	55.72	-1.35
White only, NH	44.79	42.75	4.55
All other	37.65	33.75	10.36
**Social Determinants of Health**			
Place			
Metropolitan County	45.10	44.94	0.35
Non-Metropolitan County	47.29	47.25	0.08
Education			
Graduated, College or Technical School	43.58	43.63	-0.11
Attended College or Technical School	48.45	47.74	1.47
Graduated, High School	47.38	45.71	3.52
Did not graduate, High School	47.38	43.09	9.05
Employment			
Employed	45.87	45.17	1.53
Unemployed	52.29	48.29	7.65
Retired	45.24	45.18	0.13
Unable to work	49.54	51.51	-3.98
Homemaker or student	43.29	41.12	5.01
**High risk states of Health Behaviors in past 30 days**			
14 + Days / No physical activity	46.04	45.97	0.15
Current smoker / Yes	52.10	53.44	-2.57
Binge Drinking / Yes	48.78	47.08	3.49
SRH / Fair or Poor	48.94	50.44	-3.06
14 + days/ Physical health not good	50.87	51.78	-1.79
14 + days/ Mental health not good	53.34	54.72	-2.59

Item ‘Have you experienced the death of a family member or close friend in the years 2018 or 2019? SE = Standard Error, SOGI§, CIS Gender includes ‘I think of myself as straight and not transgender.’ SOGI§, all other includes Gay /Bisexual /Something else and transgender (male to female, female to male, gender nonconforming). NH€ = non-Hispanic. SRH¥ Self-rated health 5 categories: excellent, very good, good, fair, and poor. Health behaviors reflect Healthy People 2020 target areas described in accessed April 11, 2021. https://www.healthypeople.gov/2020/topics-objectives

For 2019 BRFSS Questionnaire https://www.cdc.gov/brfss/questionnaires/index.htm; accessed May 14, 2021. RD, the relative difference between panel and weighted multiple imputation (MI). Héraud-Bousquet et al. BMC Med Res Meth 2012, http://www.biomedcentral.com/1471-2288/12/73

Table 2 shows standard errors (SE) associated with estimates for subgroups of age and categories of high-risk health states. Column 2 (Rel SE Weighted) and Column 4 (Rel SE Panel) shows the relative SE obtained from fully weighted, imputed data compared to the panel sample. The relative SE is calculated for each sample as a ratio of the SE and the prevalence estimate. The panel sample has smaller SEs. However, as with Table 1, omitting sample weights and missing responses creates biased estimates. The relative SE for the weighted MI estimates associated with age ranges from 9.34% (ages 18 to 24 years) to 3.9% (ages 65 and over). The relative differences in SE for high-risk health states ranges from 6.78% (binge drinking) to 4.33% (no physical activity for 14 or more days in a month). These Rel SE are less than 30% which makes them acceptable for public health reporting.

**Table 2:** Relative Standard Error (SE, %), Weighted-Multiple Imputed data versus Panel data, 2019 Georgia Behavioral Risk Factor Surveillance System (BRFSS)

	**Weighted, SE** *N* = 8,164,018	**Rel SE, Weighted** (%)	**Panel, SE** *N* = 4,289	**Rel SE, Panel** (%)
**Age Groups, years**				
18 – 24	3.50	9.34	2.71	7.04
25 – 34	3.21	7.36	2.20	5.16
35 – 44	3.13	6.55	2.00	4.13
45 – 54	2.63	5.48	1.83	3.80
55 – 64	2.56	5.10	1.55	3.19
65 +	1.71	3.90	1.13	2.50
**High risk states of Health Behaviors, in past 30 days**				
14 + days / No physical activity	1.99	4.33	1.24	2.69
Current smoker / Yes	3.12	5.84	2.09	4.01
Binge Drinking / Yes	3.19	6.78	2.08	4.26
SRH / Fair or Poor	2.31	4.58	1.43	2.92
14 + days/ Physical health not good	2.67	5.16	1.76	3.46
14 + days/ Mental health not good	2.68	4.90	1.88	3.52

Relative difference equals Standard error for weighted Imputed bereavement minus SE for panel data presented as a percent ratio of the SE weighted imputed

Source: https://www.cdc.gov/nchs/data/nnhsd/StandardErrors/nnhs/StandardErrors_QualityOfLifeTables.pdf

Does bereavement increase the probability of reporting high-risk health behaviors and poor quality of life? This analysis uses data from the 5,206 persons interviewed in the bereavement module. Table 3 demonstrates hypotheses testing for subgroup differences in health behaviors. This table is provided so that readers can begin to think about the application of this new item. It is not designed to present a definitive assessment of gender differences. Gender was selected for subgroup comparison because there are no missing responses to this item in the panel. Due to missing responses for the other items, imputed data was used in this demonstration. The BRFSS is cross-sectional data, so logistic regression modeling is used for this demonstration. Odds ratio and 95% confidence intervals are shown for both panel (Scenario A) and MI data (Scenario B). The odds ratios shown are adjusted for age and race because preparatory analyses show significant differences across age groups and racial groups. Within each category of health, the rows are organized to show compare 3 scenarios—bereaved with not bereaved (Model 1A,1B; 4A, 4B,7A, 7B,10A, 10B, and 13A and B), males with females (Model 2A & B, 5A & B, 8A&B, 11A&B, 14A&B), and inclusion of an interaction term (Model 3A&B, 6A&B, 9A&B, 12A&B, 15A&B). In total, there are 15 models in the table.

**Table 3:** Logistic Modeling of gender differences in effect of bereavement on health behavior states. Demonstration of Panel versus MI data. 2019 Georgia BRFSS

		**Scenario A** **Panel Sample** **N = 5,206**			**Scenario B** **Multiple Imputation and Weights (MI)** **N = 8,164,018**		
**Health, Past 30 days**	**Models**						
		**OR**_**adj**_	**CI**_**L**_	**CI**_**U**_	**OR**_**adj**_	**CI**_**L**_	**CI**_**U**_
**Binge Drinking**	**1** Bereavement	**1.37**	1.03	1.81	1.31	0.85	2.03
	**2** Gender	**2.35**	**1.81**	**3.05**	**1.94**	**1.34**	**2.79**
	**3** Bereavement*Gender	0.77	0.53	1.12	0.82	0.47	1.44
**Current smoker**	**4** Bereavement	**1.52**	**1.17**		**1.68**	**1.16**	**2.43**
	**5** Gender	**1.66**	**1.28**	**2.14**	**1.52**	**1.08**	**2.14**
	**6** Bereavement*Gender	0.74	0.52	1.06	0.78	0.46	1.31
**Self-Rated Health**	**7** Bereavement	**1.30**	**1.09**	**1.54**	**1.33**	**1.02**	**1.73**
	**8** Gender	1.11	0.92	1.33	1.13	0.87	1.47
	**9** Bereavement*Gender	**0.73**	**0.56**	**0.95**	0.92	0.61	1.39
**Physical Health**	**10** Bereavement	**1.24**	**1.01**	**1.52**	1.26	0.92	1.72
	**11** Gender	0.95	0.76	1.18	0.88	0.66	1.17
	**12** Bereavement*Gender	0.93	0.68	1.28	1.14	0.72	1.78
**Mental Health**	**13** Bereavement	**1.38**	**1.12**	**1.71**	**1.64**	**1.20**	**2.23**
	**14** Gender	**0.77**	**0.60**	**0.98**	0.83	0.60	1.14
	**15** Bereavement*Gender	1.08	0.77	1.51	1.01	0.63	1.60

*OR*_*adj*_ Odds ratio adjusted for Age and Race. *CI*_*L*_ 95% Confidence interval, lower limit, *CI*_*U*_ 95% Confidence interval, upper limit. Bold numbers indicate *p* > .10

When viewing this table, start with the challenge of studying bereavement and its potential association with binge drinking alcohol under different data scenarios and proceeds as follows. First, is there an association between bereavement and binge drinking (Model 1)? Next, is there an association between gender and binge drinking (Model 2)? Finally, an interaction term (bereavement *gender) is modeled (Model 3). What about different data scenarios—Panel (A) and MI (B)? Models A1 and B1 do not yield the same result. Model A1 yields an OR_adj_ of 1.37 and a 95% CI ranging from 1.03 to 1.81. This indicates that bereaved persons have a statistically significant likelihood of reporting binge drinking when compared to those without bereavement. Model B1 yields an OR_adj_ of 1.31 combined with a 95% CI ranging from 0.85 to 2.03, suggesting no significant association between bereavement and bingeing. What about gender differences in binge drinking? In Model A2, the OR_adj_ compares males and females; the anticipated higher risk of bingeing for men is clearly shown in both scenario A—OR_adj_ = 2.35; 95% CI 1.81 – 3.05—and scenario B—OR_adj_ = 1.94; 95% CI 1.34 – 2.79. Does gender modify the risk of binge drinking within the context of bereavement? Model 3A and 3B show that the OR_adj_ is not significantly under either data scenario. Men are not more likely than women to have an association between bereavement and binge drinking. This 3^rd^ model shows the challenge associated with evaluating bereavement and health and provides a cautionary note for thinking about gender and health effects. The results of logistic regression revealed that the CIs are wider for the weighted-MI estimates as the SEs are larger reflecting the effect of weighting and MI.

## Discussion

The goal of this study was to evaluate the performance of an item assessing recent bereavement in BRFSS—a complex sampling survey, designed to provide population numbers for use in public health planning [29, 30]. Its design includes features that account for the distribution of a state population within counties and by demographic characteristics. These features gives state and local governmental agencies the numbers needed to create cost estimates for the development of programs and their related resources. BRFSS is also used by the social science community to test hypotheses related to the social determinants of health [31].

Bereavement in a surveillance survey advances the study of bereavement health effects because a broader age group is included. Prior surveys of bereavement are limited to adults aged 50 years and older. This event can happen to anyone at any age in the life cycle. By starting at 18 years and not having an upper limit, our measurement captures bereavement earlier parts of the adult life cycle. These rates provide evidence that across all age groups, participants are willing to answer sensitive questions. However, more work is needed to evaluate the responsiveness of adolescents and children to survey items about bereavement. To gain greater details, these items can be incorporated in the Youth Risk Behavior Surveillance System (YRBSS). YBRSS monitors six types of health-risk behaviors that contribute to the leading causes of death and disability among youth and adults [32]. These behaviors are sexual behaviors, alcohol and other drug use, tobacco use, unintentional injury and violence, unhealthy diet, and inadequate physical activity. Bereavement may be a factor increasing likelihood for these risky behaviors. The YRBSS data set consists of representative samples of students typically in grades 9—12 and occurs every two years. YBRSS begins with school recruitment. School response rates range from 73 to 100%. Student response rates range from 60 to 88%. This makes YBRSS ideal for bereavement surveillance.

One challenge to the statistical use of these data is the potential for rapidly diminishing numbers in targeted subgroups. Analysts wanting to compare rates across categories of Social Determinants, Health Behaviors, and Quality of Life may not detect significant differences in the effects due to missing responses. These results show that imputation is a robust statistical method to reduce bias. This report compared estimates under two data scenarios – one without weighting and ignoring missing responses (panel) and the other using fully weighted with imputation techniques (MI). Each data scenario yielded prevalence estimates with small standard errors. One interpretation of the size of the SE is that bereavement is sufficiently common within all subgroups. Estimates from the panel data had smaller standard errors. However, when compared to MI, panel data either over- or underestimates bereavement rates. In a resource limited setting, the use of fully weighted and imputed data provides a better accounting of the numbers of affected persons and the resources needed for their care.

All the strengths and weaknesses of surveillance for recent bereavement can be seen in the analyses of bereavement and its possible association with binge drinking. Reduction in binge drinking rates – particularly for teens and young adults – is a target public health goal [30]. In BRFSS, missing responses are an issue for both items—binge drinking (11.07%) and bereavement (29.0%) Despite missing data, both panel and MI samples yield stable estimates. However, these estimates are not identical. The panel provides overestimates by 3.49% and is associated with a 34.08% relative difference in SE. This difference matters when the goal is counting the number needing care for alcohol abuse. The analyses with MI data did not show a gender difference in risk while panel data does suggest differences. Based on these results, analysts might be tempted to exclude women from binge drinking interventions because ‘*Women are less likely to binge drink*’. This could create a disparity in access to therapy for alcohol abuse by bereaved women. To avoid this bias, it is important to understand that bereavement can occur for anyone, and it is the unhealthy behavior that is the target of successful care. In short, any recently bereaved person can engage in binge drinking.

As far as is known, there are no population-based prevalence estimates for the numbers of persons reporting recent bereavement in a 24-month period that can be used for comparison. Further field testing of the bereavement item is needed to replicate these results. Without comparison data, statistical strategies and their underlying assumptions are a critical starting point for this evaluation. Measuring this recent exposure accurately and precisely is required. Population-level bereavement estimates are comparable to a flood safety risk assessment [7]. Like a flood, surveillance surveys measure bereavement within a specific time frame. Its contribution to excess numbers of persons with an associated injurious health behavior can be counted. In our demonstration of its application, the estimate of people binge drinking is 1,343,530. Within the population of binge drinkers, there are 685,517 persons (51%) who are also recently bereaved. If this association is confirmed, then any strategy to reduce the prevalence of binge drinking also requires attention to recent bereavement. Bereavement also has well described short and long-term economic consequences [33, 34]. Sometimes we forget that mortality creates orphans as well as widows. During childhood, death of a parent threatens health and economic security of surviving children well into their adult lives [35–38]. Bereavement has the potential to operate as an emerging risk factor leading to declines in current and future indices of population health.

## Conclusions

The prevalence of bereavement in Georgia is 45.38% in 2018 and 2019. Recent bereavement can be ascertained in a surveillance survey with high rates of response and small standard errors. Weighting combined with application of multiple imputation provides estimates that can be used for needs-based planning and costing. More field testing is required to replicate these results in other states, for younger individuals, and in subsequent years.

## Supplementary Information

**Additional file 1:** **AppendixTable A.** Variablesused in this analysis, 2019 Georgia BRFSS, Unweighted Panel. **Appendix Table B.** Sex, Sex-at-Birth, and SOGI Questions of theBRFSS, by Year Sex Question (Demographics Section) Sex Question (Screening Section) 2019: Are you male or female?

## Data Availability

The Centers for Disease Control and Prevention contain BRFSS data and related guidance. Data from the Georgia Bereavement Module can be obtained from Rana Bayakly; RANA.BAYAKLY@DPH.GA.GOV (Georgia, Department of Public Health).

## Abbreviations

CCA: Complete case analyses
CP: Clopper-Pearson method
GA BRFSS: Georgia Behavioral Risk Factor Surveillance System
HRS: Health and Retirement Survey
KG: Korn-Graubard method
MAR: Missing at random
MCAR: Missing completely at random
MI: Multiple Imputation
MICE: Multiple imputation by chained equations
MNAR: Missing not at random
QOL: Quality of Life
SDOH: Social Determinants of Health
SE: Standard Error
SOGI: Item in the GA BRFSS, Sexual Orientation and Gender Identity. Categories – SOGI CIS Gender ‘I think of myself as straight and not transgender.’ SOGI All others: Includes Gay / Bisexual/ Something else and transgender (male to female, female to male, gender nonconforming)
US: United States
RD: Relative Difference
RR: Response rate
YRBSS: Youth Risk Behavior Surveillance System
