# Supplementary material for: Measuring bereavement prevalence in a complex sampling survey: the 2019 Georgia Behavioral Risk Factor Surveillance System (BRFSS)
Source: BMC Med Res Methodol. 2023 Jun 13;23:138. doi: 10.1186/s12874-023-01917-5 (PMC10262108; doi:10.1186/s12874-023-01917-5)
Supplement: Supplementary file 1 — Additional file 1: AppendixTable A. Variablesused in this analysis, 2019 Georgia BRFSS, Unweighted Panel. Appendix Table B. Sex, Sex-at-Birth, and SOGI Questions of theBRFSS, by Year Sex Question (Demographics Section) Sex Question (Screening Section) 2019: Are you male or female? [file 12874_2023_1917_MOESM1_ESM.pdf]

Appendix Table A: Variables used in this analysis, 2019 Georgia BRFSS, Unweighted Panel

Variable	Complete Response, N	Complete %	Missing Response, N	Missing %
**Bereavement item***: Loss of family or friend in 2018 or 2019.	5,206	70.79	2,148	29.21
**Demographics**				
Gender	7,354	100.00	0	0
SOGI[§]	5,443	74.01	1,911	25.99
Age	7,354	100.00	0	0
Race /ethnicity	7,180	97.63	174	2.37
**Social determinants**				
Educational attainment	7,319	99.52	35	0.48
Metropolitan Statistical Area, residence	7,354	100.00	0	0
Employment status	7,202	97.93	152	2.07
**Health Behaviors**				
Physical activity in past month?	6,780	92.19	574	7.81
Smoking status	6,847	93.11	507	6.89
At least one drink of alcohol in past 30 days?	6,796	92.41	558	7.59
Multiple drinks on one occasion	6,540	88.93	814	11.07
Self-rated health	7,330	99.67	24	0.33
Physical Health not good, days in past month	6,802	92.49	552	7.51
Mental Health not good, days in past month	6,799	92.45	555	7.55
**Complete information above 15 variables**	4,289	58.32	3,065	41.68

Note: 'Don't know', 'Refused' and 'Blank' equal missing. *New 2019 BRFSS item '*Have you experienced the death of a family member or close friend in the years 2018 or 2019*? * SOGI[§]: Sexual Orientation and Gender Identity. Module 29, two questions '*Which of the following best represents how you think of yourself? Do you consider yourself to be transgender?* Health behaviors reflect Healthy People 2020 target areas described in https://www.healthypeople.gov/2020/topics-objectives ; Accessed April 11, 2021. For all items see 2019 BRFSS Questionnaire https://www.cdc.gov/brfss/questionnaires/index.htm; Accessed May 14, 2021.

Appendix Table B:
Sex, Sex-at-Birth, and SOGI Questions of the BRFSS, by Year Sex Question (Demographics Section)
Sex Question (Screening Section) 2019: Are you male or female?

SOGI Optional Module 2018-2019:

(For male respondents)
Which of the following best represents how you think of yourself?
1 = Gay
2 = Straight, that is, not gay
3 = Bisexual
4 = Something else
7 = I don't know the answer
9 = Refused Ask if Sex= 1.
(For female respondents)
Which of the following best represents how you think of yourself?
1 = Lesbian or Gay
2 = Straight, that is, not gay
3 = Bisexual
4 = Something else
7 = I don't know the answer
9 = Refused
Do you consider yourself to be transgender?
1 Yes, Transgender, male-to-female
2 Yes, Transgender, female-to-male
3 Yes, Transgender, gender nonconforming
4 No

Source: https://www.cdc.gov/brfss/data_documentation/pdf/BRFSS-SOGI-Stat-Brief-508.pdf .
Accessed March 24, 2022.
